# On the Medicinal Effects of the Carbonat of Potass, or Salt of Tartar, in Puerperal Fevers

**Published:** 1800-02

**Authors:** 


### *On the Medicinal Effects of the Carbonat of Potaſs, or Salt of Tartar, in Puerperal Fevers; by Citizens* Allan, Deyeux, Gilbert, *and* Lafisse.

[ Continued from our laſt Number, p. 80 and 81. ]

A Long experience having ſufficiently convinced Cit. Guinot of the advantages of his method, he preſcribes the carbonat of potaſs, as a preſerative againſt puerperal fevers in large hoſpitals, where it attacks every tenth woman in child-bed. He alſo recommends the prudent uſe of ammonia for the purification of the air.

Such is the ſubſtance of the firſt part of Cit. Guinot's Memoir, on which we intend to make ſome reflections, not with a view to diminiſh its value, but becauſe the moſt plauſible opinions ought not to be admitted in medicine, till they have been ſubjected to ſcrupulous inveſtigation.

1. It is now well known, that in general all urine, even that of the moſt healthy individuals, contains a quantity of acid. The uric, phoſphoric, and benzoic acids, are commonly found in it, in different proportions, in a more or leſs diſengaged ſtate. Hence, no particular concluſion can be drawn, drawn, as the urine of a woman, attacked with puerperal fever, reddened the tincture of turnsol; and, though the tinge appeared deeper than that imparted by the urine of others, this difference might arise from circumstances unconnected with the disease.

2. All women in child-bed exhale an effluvium similar to that of sour milk, particularly when they have full breasts, and are kept very warm. The humour they transpire is then so evidently acid, that blue paper when applied to the skin, in a few minutes, acquires a red colour; and yet, all lying-in women are not afflicted with puerperal fever.

3. The experiments made by Cit. Guinot on different kinds of cheese, by dissolving them with potass, do not prove that the real effect of this medicine in the disease abovementioned, is that of saturating the superabundant acid: for, what a quantity of alkali would it not require, to absorb all the acid, which he supposes to be extended through the general mass of humours, and in their deposed matter? And is it not probable, that the small quantity of alkali which can be taken into the stomach, is first neutralized by that organ, or, at least, a considerable part of it, by the gastric juice?

4. It is probable, that the milk which accumulates in the abdomen, or is diffused into other cavities, retains its natural fluidity without having undergone any change. In fact, if it were first coagulated by an acid, how is it possible that it could flow over the membranes, or circulate in the cellular texture? The milk changes its nature only after the formation of the sediment. Having undergone this change, it turns acid, and soon becomes putrid. Hence, the acid is, in this instance, the effect and not the cause of the disease.

We may farther convince ourselves of this fact, by attending to the circumstances which commonly attend puerperal fever. Sudden chillinefs, excessive joy, tendency to anger, peevishness, fright, and infected air, are often productive of that disease; and yet, none of these causes appear to indicate the existence of an acid, nor to contribute to its formation.

The most constant symptom of puerperal fever, is the dependent and flaccid state of the breasts. If the milk has previously been determined towards these organs, it suddenly disappears on the attack of the disease; but, if the attack of fever commences sooner, the milk does not appear. The real cause then is metastasis of the milk, which has a tendency to deposite particles of a caseous and serous nature, either in the abdomen, as is most frequently the case; in the breast and head, as Bordeu has witnessed; or even in the liver, as has been observed by Doublet, or in the external cellular membrane. The symptoms fymptoms of inflammation, or putrefaction, which fometimes intervene, are only fecondary, and do not conftitute the principal character of the difeafe. It may, as Mr. Whytt imagines, be complicated with the hofpital fever; and, perhaps, from this reafon it is fo common in large hofpitals. How can the metaftafis of the milk, which fuddenly difappears from the breafts, be fuppofed to depend on a fuperabundance of acid? If this acid previoufly exifted, the milk, inftead of efcaping fuddenly, would coagulate in the breafts, where it would occafion obftructions difficult to be refolved; but it would not produce puerperal fever, as is proved by experience.

However the theory of Cit. Guinot may be received, his practice is advantageous; by ufing the carbonat of potafs, either to diffolve the clotted milk, by neutralizing the acid which may actually exift there, or by its action on the organs of perfpiration; or, laftly, by inducing other ufeful crifes. This will appear from the obfervations contained in the fecond part of the Memoir, from which we fhall give an extract.

[ To be continued in our next Number. ]

---

Cit. Alibert read a differtation on the cholic that prevailed at Madrid. His principal object was to prove that difeafes, though perfectly analogous, ought not to be treated in the fame manner, in different climates. The cholic of Madrid, notwithftanding its complete fimilarity to that of Poitou, does not yield to the fame remedies; and the draftic laxatives employed in the hofpital La Charité de Paris, has proved very detrimental to the patients at Madrid. He has added to this memoir, fome obfervations on the ftate of the medical art in Spain.—Rapport Général de la Société Philomatique de Paris, vol. ii. p. 82.

---

Citizens Alibert, Fourcroy, and Hallé, made fucceffive reports on the treatment of a man who was covered with a fpecies of leprofy. This patient was manifeftly relieved by frictions of oxygenated pomatum, fo that, when the fcabby parts were anointed, the incruftations fell off, and the extreme itching which he previoufly felt, was confiderably abated. Several other fatisfactory reports have been prefented to the Society, on the effects of this remedy, in fimilar cafes.—*Ibid.* 83.

---

Citizens Alibert and Dumeril have continued their experiments with medicinal fubftances applied in frictions. Several children, whofe abdominal vifcera were confiderably fwelled, were purged by rhubarb, and a combination of fcammony with the the gastric juice of an owl was administered by friction. Another child, three years old, who laboured under symptoms from which hydrothorax was apprehended, discharged a large quantity of urine, in consequence of frictions made with an ointment consisting of the squill macerated in the gastric juice of a dog, and mingled with hogs-lard. The bark was also administered in a similar manner, which prevented fever in some patients, and diminished or changed it in others.—*Ibid.* 83, 84.

---

Cit. Faivre transmitted a memoir on the effect of opium, and the super-oxygenated muriat of mercury, administered by frictions in the treatment of venereal diseases. By this process he cured an exostosis which he had in vain attempted to reduce by internal remedies, as the delicate nature of the disease precluded the administration of the latter.—*Ibid.* p. 84.

---

Cit. Larrey presented a memoir on the disorganization of several of the abdominal viscera, by the kick of a horse, without lacerating the skin. The patient felt an excruciating pain throughout the abdomen; this agony increased, was accompanied with vomitting, and succeeded by death, in about fifteen hours. On opening the body, a part of the transverse colon was found suffused with blood. The gall-bladder was full and distended, part of the liver swollen, the mesocolon divided, and the second curvature of the duodenum was partly torn; there was also a discharge of blood and alimentary matter.—*Ibid.* p. 85, 86.

---

The same member informed the Society, that he had recently performed an operation upon a young woman, about twenty-five years of age, whose genital parts were imperforate. The skin was continued on the pubis, leaving only a small aperture for the passage of the urine. She had never menstruated. Cit. Larrey opened the external skin, which covered the pubis, and thus restored the parts to their natural state.—*Ibid.* p. 86.

---

Cit. Pajot des Charmes, read an essay on the effect of alkaline substances employed in the treatment of *fistulæ lachrymales*. He was induced to report his experiments on this subject, in consequence of his having observed, that the use of a piece of linen, newly washed, and thus saturated with an alkaline lye, after several applications, effectually relieved the symptoms of that disease. This simple method, according to his experience, uniformly and perfectly removed the complaint.—*Ibid.*

*Pharmaceutical*

